# 

**Published:** 1893-06-03

**Authors:** 


No. 349, Vol. XIV.—June 3rd, 1893.

[Registered at the General Post Office as a Newspaper for transmission in the United Kingdom and Abroad.]

WITH EXTRA NURSING SUPPLEMEN

Per Annum, 8s. 6d., post free.

Monthly Parts, 6d.; post free, 8d.

Ditto per Annum, 8s., post free

### THE HOSPITAL

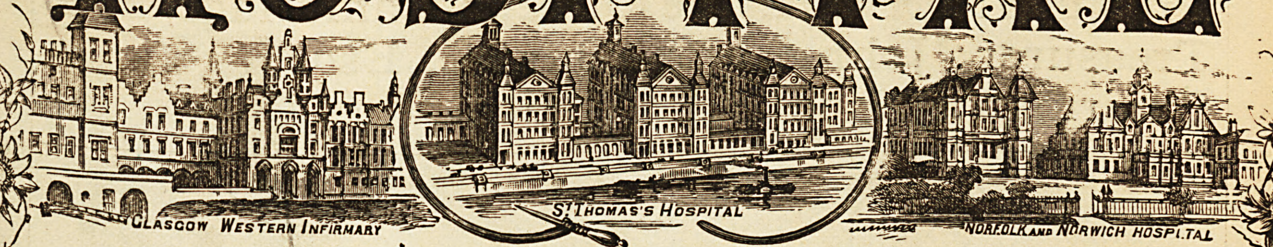

WITH EXTRA NURSING SUPPLEMENT.

No. 349. Vol. XIV.] SATURDAY, JUNE 3RD, 1893. [TWOPENCE.

